# The effect of two preparation designs on the fracture resistance and marginal adaptation of two types of ceramic crowns using CAD/CAM technology (In vitro study)

**DOI:** 10.1186/s12903-024-04742-4

**Published:** 2024-09-11

**Authors:** Akram Moamen Ashour, Mohamed Mahmoud El-Kateb, Amir Shoukry Azer

**Affiliations:** Division of Fixed Prosthodontics, Department of Conservative Dentistry, Alexandria University, Alexandria, Egypt

**Keywords:** Fracture resistance, Marginal adaptation, Vertical preparation, Modified vertical preparation, CAD/CAM materials

## Abstract

**Background:**

Recently, prosthodontic approaches involve more conservative procedures that include less invasive finish line preparations that use less ceramic thickness.

**Aim of the study:**

This in vitro study aimed to evaluate the effect of vertical preparation and modified vertical preparation designs on the marginal adaptation and fracture resistance of two types of ceramic crowns using CAD/CAM technology.

**Materials and methods:**

Two typodont maxillary first premolars were embedded in acrylic resin. Forty positive replicas of epoxy resin dies were used that were divided into two groups depending on the preparation design (*n* = 20); Group V (Vertical): dies with feather edge finish line and Group MV (Modified vertical): dies with feather edge finish line, where a reverse shoulder of 1 mm depth was placed on the buccal surface 1.5 mm from the occlusal surface. Each group was further subdivided into two subgroups according to the type of ceramic material (*n* = 10): Subgroup Va and subgroup MVa for lithium disilicate (e.max CAD) and subgroup Vb and subgroup MVb for zirconia (zolid ht+). Crown restorations were made with CAD-CAM technology. The marginal adaptation was assessed using a stereomicroscope both prior to cementation and after cementation and aging. Fracture resistance was tested with a universal testing machine, and the data were statistically analyzed.

**Results:**

Marginal adaptation showed no significant differences between subgroups before or after cementation and aging. Three-way ANOVA indicated that preparation design (*p* = 0.516) and material (*p* = 0.269) had no significant effect, but cementation had a significant effect (*p* < 0.0001) on the marginal adaptation. According to two-way ANOVA test, Subgroup (MVb) showed the highest result followed by subgroup (Vb) and subgroub (MVa) and the least was subgroub (Va). Fracture modes showed no significant differences among the subgroups (*p* = 0.982).

**Conclusions:**

Marginal adaptation of lithium disilicate and zirconia crowns remained clinically acceptable regardless of preparation design. While the modified vertical preparation with a reverse shoulder notably enhanced the fracture resistance of both materials, with zirconia demonstrating superior fracture resistance compared to lithium disilicate with average values exceeding premolar biting force.

## Background

Preparing teeth for prosthetic crowns has traditionally been believed to be invasive because it causes irreparable loss of tooth hard tissue [1]. In general, crown tooth preparation designs are classified into two types: horizontal preparation design with finish line and vertical preparation without finish line [2].

For all-ceramic crown restoration, the horizontal preparation with shoulder and chamfer finish lines has long been considered the ideal preparation design. On the other hand, these margins are invasive as they remove vital tooth structure which is needed for esthetic and biological considerations [3]. The vertical preparation is the most conservative margin preparation design. This technique is suggested for periodontally compromised teeth, vital teeth in youths, and endodontically treated teeth [4].

However, due to the vertical preparation’s shortcomings, shoulder preparation became the gold standard in the academic community [5]. Overhangs, inability to exert control over the marginal seal, over-contouring, unpredictability of tissue healing, and extra cement removal challenges are only a few examples. Furthermore, the difficulty in detecting these thin tapered edges according to data acquired from gingival tissue by laboratory technicians and the sensitivity of the restorations to chipping fracture has always restricted the option to choose the vertical preparation method [6].

Recently, the availability of different ceramic materials and fabrication procedures can permit the use of a minimally invasive conservative approach with enhanced predictability [7]. Ceramic systems are rapidly improving making it possible to treat teeth in both the posterior and anterior areas, with the main objective of restoring shape, esthetic, and function without the use of metal. Intraoral scanners (IOSs) and CAD/CAM technology have enabled the manufacture of metal-free restorations, providing the ability to improve the biomimetic and esthetic results of restorations [8, 9].

Lithium disilicate and Polycrystalline zirconium dioxide ceramics are commonly used in restorative indirect restorations due to their superior aesthetic and mechanical characteristics [10]. Zirconia offers great biological compatibility and mechanical properties, with a flexural breaking strength of 900–1200 MPa, and sufficient optical features, allowing all ceramic restorations to be used in posterior areas that require more fracture resistance [11]. Although outstanding strengths for zirconia core material have been recorded, some research on veneering ceramics has reported fractures [12, 13]. Zirconia crown restorations are made monolithically without veneering to avoid fracture and simplify manufacturing procedures. Several studies have found that monolithic zirconia crowns are more fracture-resistant than standard veneered counterparts [14, 15]. The glass-ceramics group promises to deliver the finest clarity and esthetic features. Even though advanced lithium disilicate has enhanced mechanical properties with flexural strengths ranging from 300 to 400 MPa, this ceramic group provides a better adhesive bond to the tooth when used with adhesive resin cement, allowing those who practice to carry out more conservative preparation designs [16].

However, using high-strength ceramic materials can decrease the invasiveness of tooth preparation by using a minimum preparatory design [17, 18]. It is essential to evaluate the mechanical performance of all-ceramic material with knife edge margin as the fracture strength of all-ceramic restorations can be affected by a variety of factors, including the margin design [19]. On the other hand, the extended clinical efficacy of dental restorations is influenced not only by, aesthetic quality, biocompatibility and mechanical properties but also by marginal adaptation [20, 21].

The goal of this study was to compare the impact of the vertical preparation and modified vertical preparation on the marginal adaptation and fracture resistance of crowns manufactured from two different ceramic materials: Lithium disilicate and zirconia crowns using CAD/CAM technology. The null hypothesis was that there will be no difference in marginal adaptation and fracture resistance between the tested groups.

## Materials and methods

An overview of the materials used in the present study is listed in Table 1.

**Table 1:** A general description of the materials with their manufacturers and compositions

Material	Product name	Composition	Manufacturer
Lithium Disilicate	IPS e.max CAD	57–80% SiO_2_, 0–13% K_2_O, 0–5% Al_2_0_3,_ 11–19% Li_2_O, 0–8% ZrO_2_.	Ivoclar Vivadent, Schaan, Liechtenstein
Zirconia	Ceramill zolid ht+	≥ 99.0% (Yttrium oxide (Y2O3) + Zirconium dioxide (ZrO2) + Hafnium oxide (HfO2)), ≤ 0.5% Al2O3, ≤ 1% other oxides	Amann Girrbach AG, Austria
Hydrofluoric acid	Porcelain Etchant	9.5% hydrofluoric acid	BISCO-Schaumburg, USA
Porcelain primer	Porcelain primer	30–50% Ethanol, 30–50% Acetone, Silane 1–5%	BISCO-Schaumburg, USA
Zirconia primer	Z-Prime Plus	75–85% Ethanol, 5–10% Bisphenol A Diglycidylmethacrylate, 5–10% Hydroxyethyl Methacrylate, 1–5% MDP	BISCO-Schaumburg, USA
Universal adhesive resin luting cement	Duo-Link Universal	Ytterbium Fluoride, Urethane Dimethacrylate, Ytterbium Oxide-Silica, Tetrahydrofurfuryl Methacrylate,, Bisphenol A Diglycidylmethacrylate, Trimethylolpropane trimethacrylate.	BISCO-Schaumburg, USA

### Preparation of the ivory teeth

**Fig. 1:**
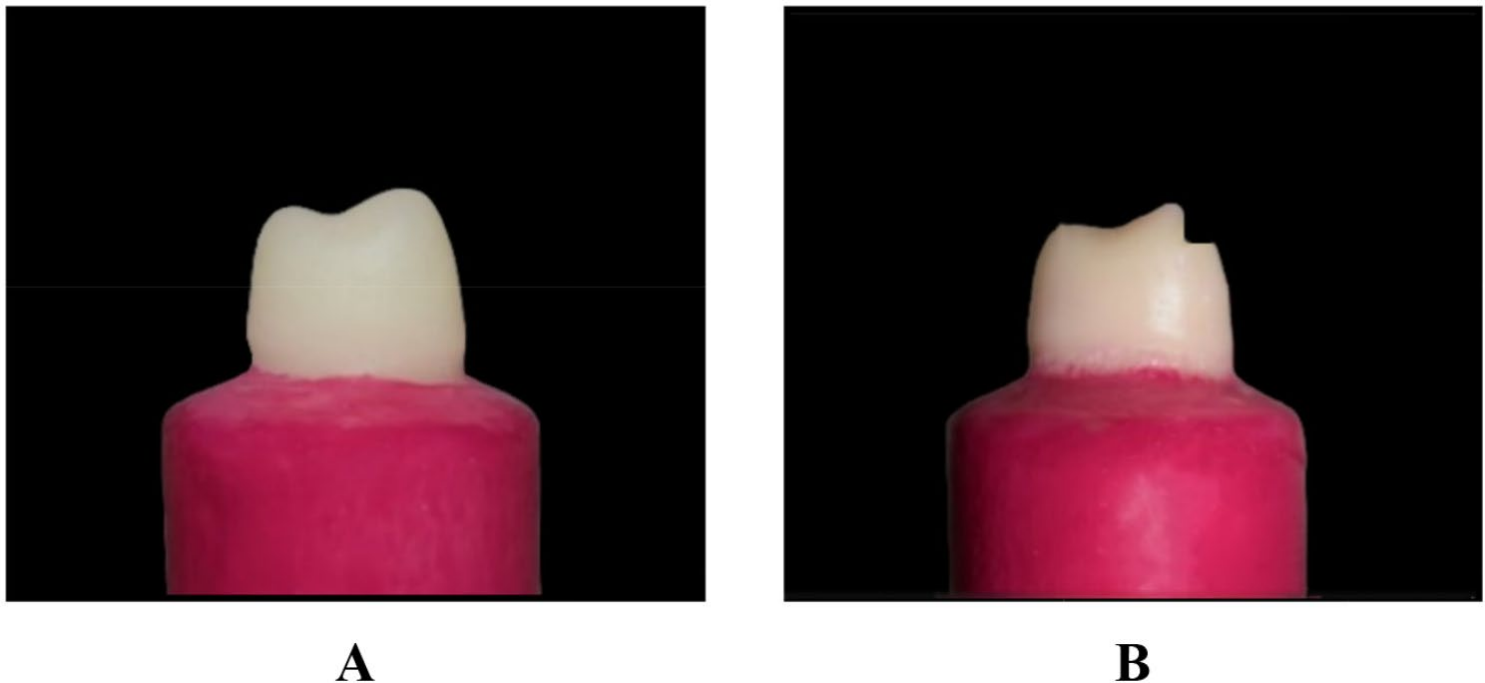
**A** Vertical preparation. **B** Modified Vertical preparation

**Fig. 2:**
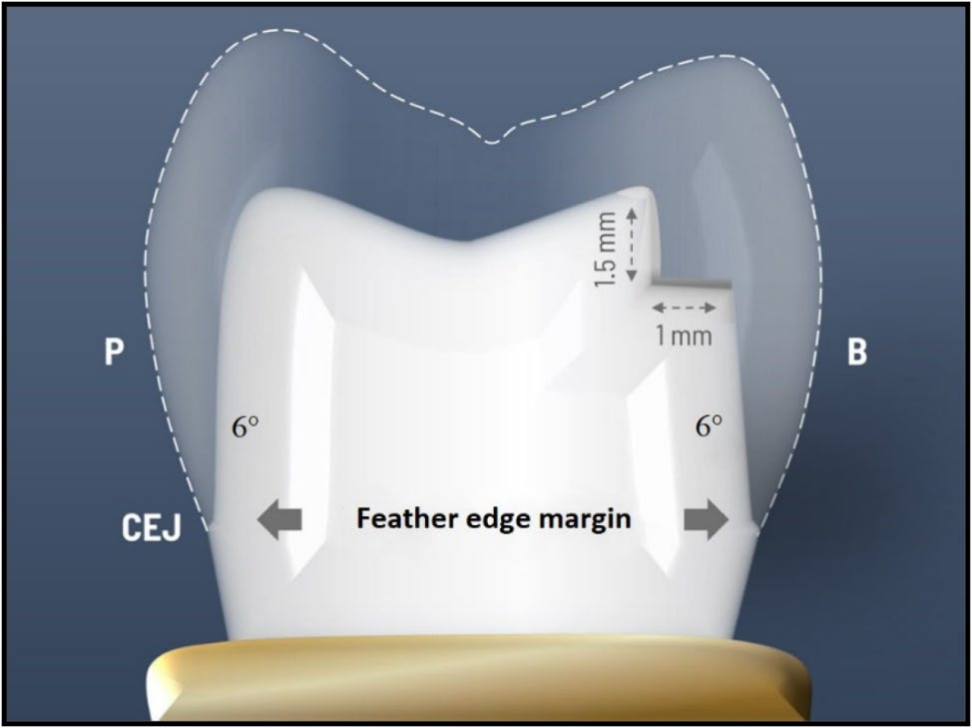
Illustration showing the modified vertical preparation design

Two typodont maxillary first premolar teeth were placed in an acrylic resin block (Acrostone, Egypt), showing only the crown and 2 mm apical from the cementoenamel junction (simulated bone level). For standardization purposes, an optical impression of the typodont tooth was done by an intraoral scanner (Sirona Dental System, GmbH, Bensheim, Germany) to consider and standardized the size of all the milled crowns after preparation, then a modified surveyor (Saeshin, Daegu, Korea) was used during the preparation with a water-cooling high-speed handpiece (Apple Dental, Hunan Jinme, Hong Kong) that was attached to the surveyor’s vertical arm. For the two teeth preparation; by using a round-end tapered diamond bur, the occlusal reduction was done by reducing the occlusal surface by 1.5 mm on a non-functional cusp and 2.0 mm on a functional cusp. For axial preparation; a safe-ended tapering round diamond bur (851 314 012, Komet, Germany) was used with a 6° convergence angle was used to establish a feather edge margin finish line that was 1 mm above the CEJ (Fig. 1A). For the tooth with modified vertical preparation, a flat-end diamond fissure bur (8372P 314 023, Komet, Germany) was used to place a 1 mm deep reverse shoulder on the buccal surface and 1.5 millimeters from the occlusal surface. (Figures 1B and 2)

### Fabrication of the epoxy resin dies

Additional silicone duplicating material was used to take impressions (Dupliflex, Protechno, Spain) of the two prepared ivory teeth. For 30 s, the two silicone duplicating material components were mixed in the same ratio (1:1), as directed by the manufacturer until a uniformly colored mixture was created. The mixture was then placed in a plastic ring and positioned on the two prepared ivory teeth until the material was set. After that, each mold of the two different preparation designs was subsequently filled 20 times with a non-shrink epoxy resin (Kemapoxy 150, CMB, Egypt) and left for 24 h. Subgroups’ titles were carved on the underside of the dies for easy identification.

### Grouping

All epoxy dies were classified based on the preparation design into two groups (*n* = 20 for each group).

- **Group V**: Twenty Epoxy dies prepared with vertical preparation design. **Group MV**: Twenty Epoxy dies prepared with modified vertical preparation design.
  Depending on the used ceramic system, each group was subdivided into two subgroups. (*n* = 10).
- **Subgroups Va**: Ten crowns were fabricated from e.max CAD lithium disilicate for epoxy resin dies that were prepared with vertical preparation design. **Subgroups Vb**: Ten crowns were fabricated from zolid ht + zirconia for eoxy resin dies that were prepared with vertical preparation design. **Subgroups MVa**: Ten crowns were fabricated from e.max CAD lithium disilicate for epoxy resin dies that were prepared with modified vertical preparation design. **Subgroups MVb**: Ten crowns were fabricated from zolid ht + zirconia for epoxy resin dies that were prepared with modified vertical preparation design.

### Fabrication of the restorations

The following fabrication steps were done following the manufacturer’s instructions: every epoxy resin die has been scanned by an intraoral scanner (Sirona Dental System, GmbH, Bensheim, Germany) to obtain an optical impression, full contour anatomical monolithic crowns resembling upper first premolars were designed by CAD/CAM software (Exocad dental DB), designs were done using the data that were taken from the optical impression that has been done to the unprepared typodont tooth. The thickness of all crowns was set at 1.5 mm on a non-functional cusp and 2.0 mm on a functional cusp occlusally, 0.8 mm axially with a cement space of 50 μm for the axial and occlusal surfaces. After designing, the STL file was transmitted to the milling unit for milling. A computer-controlled 5-axis milling unit (ED5X, Emar Mills, Egypt) was used for the milling procedure.

Lithium disilicate (IPS e.max CAD; IVOCLAR VIVADENT AG) crowns were produced by wet milling. Then the milled crowns were glazed with (FLUO Ivocolor glaze paste; Ivoclar Vivadent, Liechtenstein). The crystallization process was carried out according to the manufacturer’s recommendation using (Programat P310, Ivoclar Vivadent AG, Schaan/ Liechtenstein) at 820◦C for 20 min.

Zirconia crowns were milled out of zirconia blank (Ceramill zolid ht+; Amann Girrbach AG). The milled crowns were then sintered in a sintering furnace (TABEO-1/M/ZIRKON-100, MIHM-VOGT GmbH & Co. KG, Germany) at 1450 °C following the manufacturer’s instructions. During the fire cycle, the temperature progressively raised from ambient temperature to the sintering temperature (1450 °C) in 30 min. The furnace maintained the sintering temperature for nine hours, after which it began to cool down, reaching 1140 °C in eight minutes. After being removed from the furnace, the specimens were allowed to cool down to room temperature. At a 10 mm distance for 10 s, the intaglio surfaces of zirconia crowns were sandblasted with 50 μm alumina particles at 2 bar air pressure [22].

### Bonding

For e.max CAD crowns, the crowns’ intaglio surfaces were etched using hydrofluoric acid 9.5% (BISCO’s porcelain etchant; BISCO-Schaumburg, USA) for 20 s, and thoroughly rinsed with running water and cleaned with air/water spray then dried, then one coat of Silane primer (BISCO’s porcelain primer; BISCO-Schaumburg, USA) was applied by a small brush for 60 s then dried with a gentle jet of air. For zirconia crowns (Ceramill zolid ht+; Amann Girrbach AG.), two coats of zirconia primer (BISCO’s Z-Prime plus) were applied by a small brush into the intaglio surfaces of the crowns, left for 30 s and then dried by a gentle jet of air. For all crowns, an automix tip was used to apply dual-cured luting resin cement (Duo-Link Universal adhesive resin cement-BISCO-Schaumburg, USA) to the crown’s intaglio surfaces then placed onto its corresponding dies using static finger pressure. To maintain pressure during curing, all specimens were maintained under a fixed force of 5 kg using a specially developed static load device before and during curing for 10 min. Light-emitting diode (LED) curing light was used for 4 s over the crown edges to facilitate excess cement removal using a scalpel blade, followed by 40 s for each surface to achieve complete polymerization.

### Aging

**Fig. 3:**
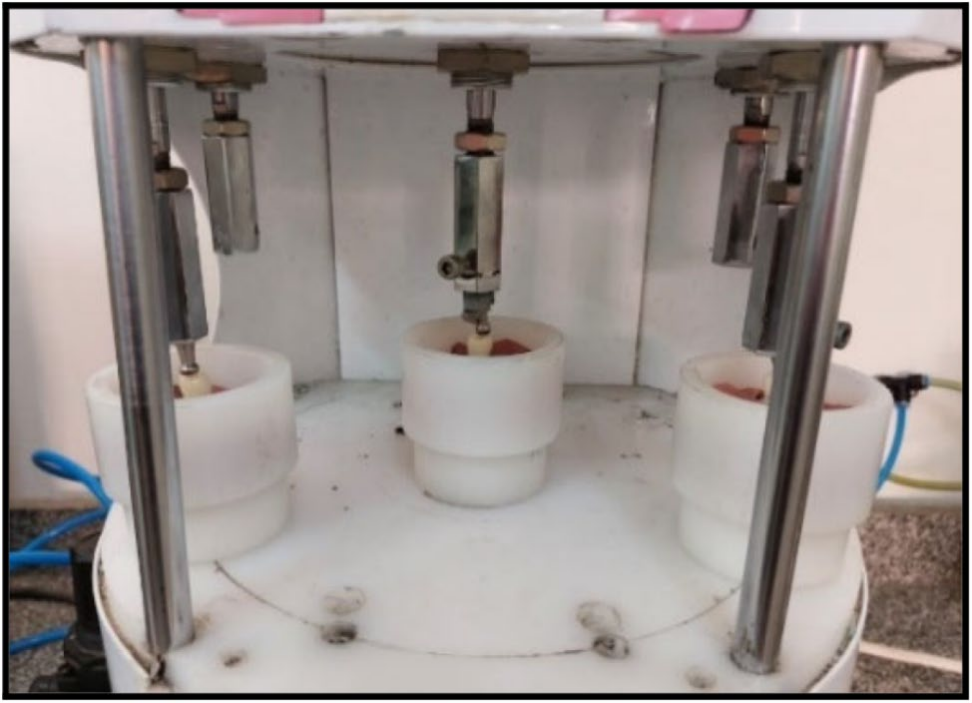
Specimen under cyclic loading

To replicate six months of clinical service, each specimen was subjected to 5000 thermal cycles. The thermal cycles were carried out mechanically as the specimens being transferred between two temperature-controlled water baths (between 5 °C and 55 °C), with a dwell time of 30 s in each bath and a relaxation period of 15 s in the air between baths) [23]. All specimens were then subjected to 120,000 mechanical loading cycles [23]. A custom-made load cycling machine with a specific design was used to carry out the cyclic loading. Stainless steel styluses, 4 mm in diameter were used to form a spherical contact surface with the occlusal surface of ceramic crowns (Fig. 3).

### Tests

**Fig. 4:**
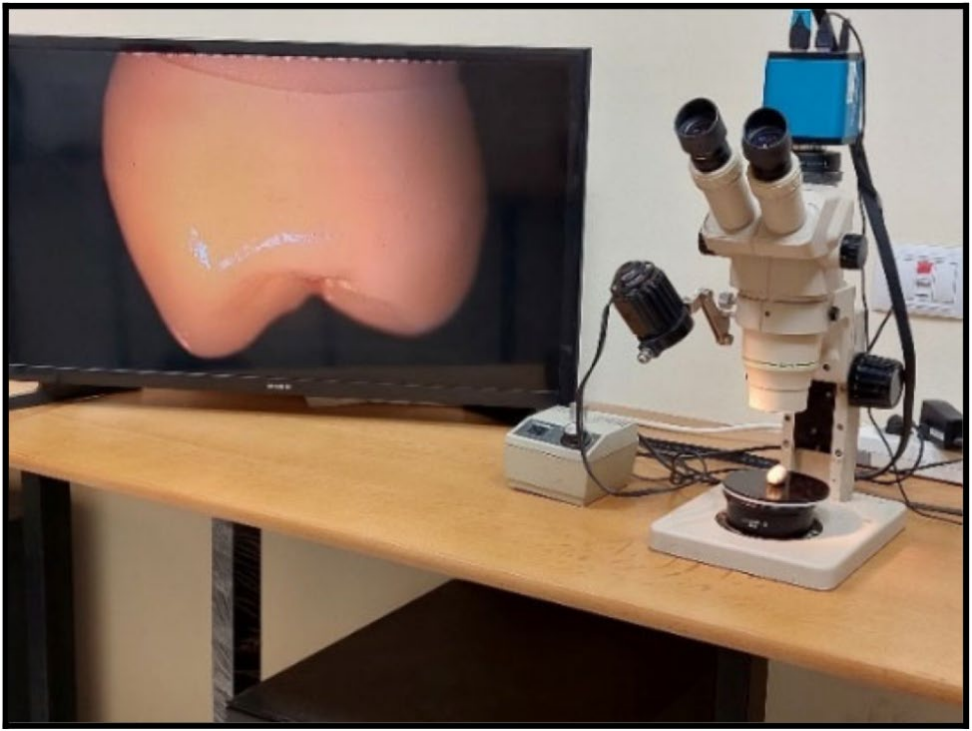
Evaluation of marginal adaptation by stereomicroscope

**Fig. 5:**
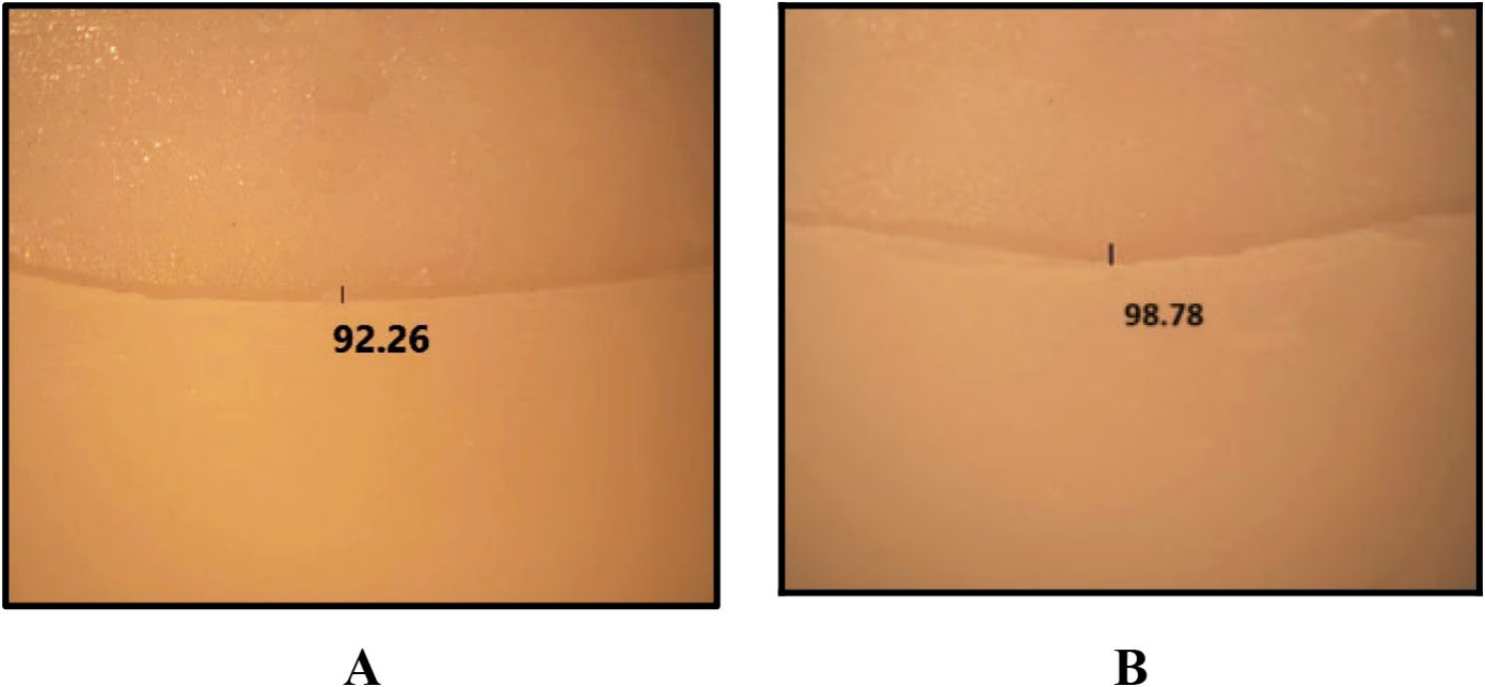
Marginal gap measurement: (**A**) Before cementation. (**B**) After cementation

**Fig. 6:**
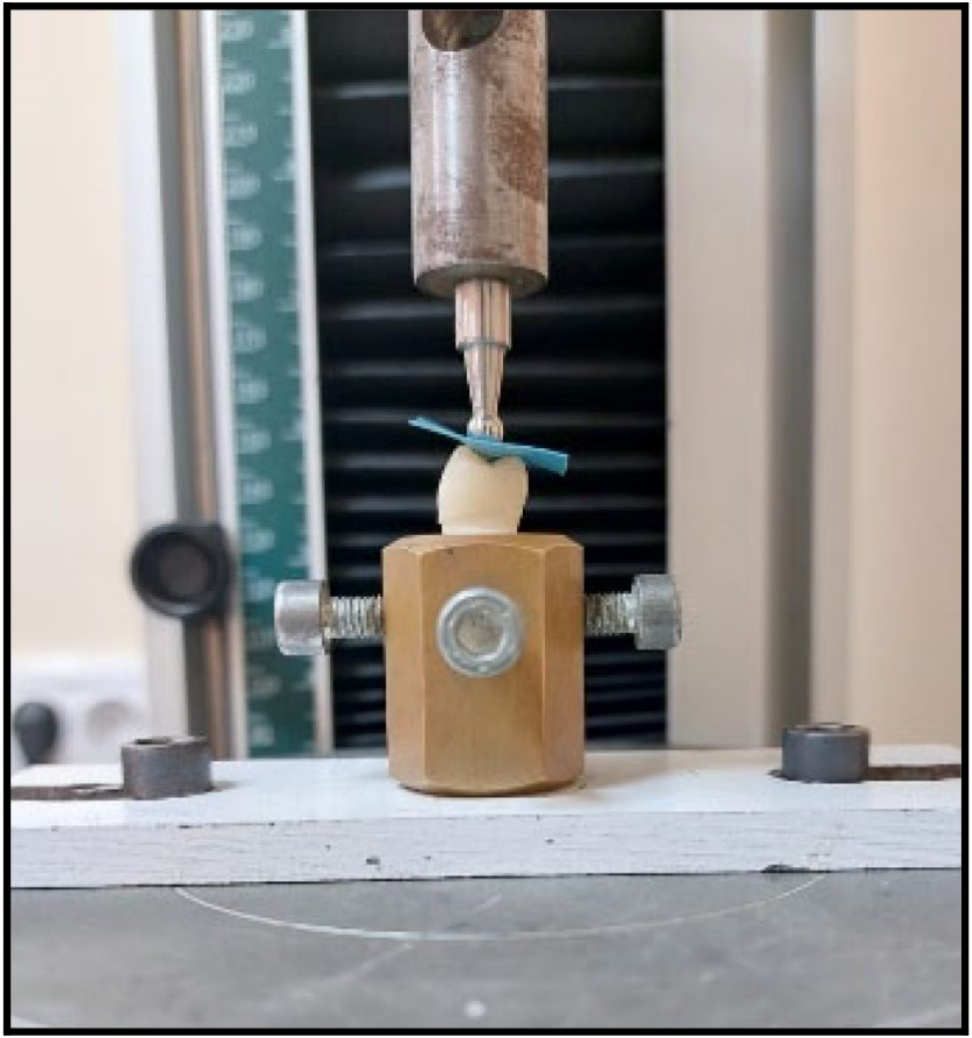
Fracture resistance test

A. marginal adaptation evaluation before cementation To evaluate the marginal fit of the crown restorations in the four studied subgroups before cementation, the cervical marginal gaps of all specimens were measured by using a stereomicroscope (Olympus SZ1145 Trinocular Stereomicroscope) (Fig. 4). A custom-made metal jig was the tool of choice to hold and stabilize the samples during the measurement of the cervical vertical marginal gap. One centralized point on each surface of each specimen (mesial, distal, buccal, and palatal) was measured at 40X magnification. Images were taken by a mounted digital camera (DP10, Olympus, Japan) (Fig. 5A). For analysis, images were uploaded into the computer software (Image J.JS V0.5.6). The data was collected for statistical analysis, and all specimens’ marginal adaptation parameter in microns was obtained.
B. marginal adaptation evaluation after cementation and aging After cementation and aging, the marginal gap of tested specimens was measured by stereomicroscope with the same magnification and at the same points (Fig. 5B). The final data was collected, and processed, and statistical analysis was performed.
C. Fracture resistance evaluation After aging and cementation, Measurements of fracture resistance were carried out using a universal testing machine (5ST, Tinius Osen, England). Each sample was attached to the universal testing machine’s base. A vertical compressive force was applied perpendicular to the occlusal surface of each specimen by a 4 mm stainless steel ball stylus that was fixed to the device’s upper arm with a cross-head speed of 1 mm/min [24] (Fig. 6). A polyurethane sheet was positioned between the stylus and the specimen to prevent undesirable contact and evenly distribute the applied force. The failure loads were measured in Newton for each specimen as the load was gradually increased till fracture. The data for all specimens were collected for statistical analysis and the mean value in Newton was calculated.
D. Assessment of mode of failure The modes of fracture of all specimens were considered and classified visually according to Burke’s classification [25]. (Table 2).

**Table 2:** Burke’s classification of modes of crown fracture

Modes of fracture	Description
I	The crown has a minor fracture or crack.
II	Less than half of the crown lost
III	Crown fracture through midline (half of the crown lost or displaced)
IV	More than half of the crown lost
V	catastrophic fracture of the tooth and/or crown

### Statistical analysis

The normality of variables was checked using Shapiro Wilk test and Q-Q plots. Both variables followed a normal distribution pattern, thus, values were summarized using mean, standard deviation and 95% confidence interval (CI). Percent change in marginal adaptation was calculated according to the following formula: [(values after cementation and aging – values before cementation)/values before cementation] x 100. It was found to be not normally distributed, therefore, median, inter quartile range (IQR), minimum and maximum were used for data presentation.

Two-way analysis of variance (ANOVA) was used to assess the main influence and the interaction between preparation design and material on fracture resistance. Also, Three-way analysis of variance (ANOVA) was employed to assess the main effect of and the interaction between preparation design, material, and cementation on marginal adaptation. Kruskal Wallis test followed by Dunn’s post hoc test with Bonferroni correction was used to compared percent change in marginal adaptation. All tests were two-tailed, with a significance level of p value ≤ 0.05. Data was analyzed using IBM/SPSS version 23 for windows, Armonk, NY, USA.

## Results

### Marginal adaptation

Results of the marginal adaptation evaluation before cementation and after cementation and aging measured in (µm) are shown in (Table 3). Before cementation (Fig. 6A), the mean ± SD of the marginal gap was the highest among (Va) subgroup (93.18 ± 1.55 μm) followed by (MVa) (92.99 ± 1.13 μm). Followed by (Vb) and (MVb) subgroups (92.81 ± 1.66 μm and 92.99 ± 1.13 μm, respectively). After cementation and aging (Fig. 6B), all marginal gap values were increased significantly with the highest values reported for e.max CAD whether made with vertical or modified vertical preparation (99.72 ± 3.34 μm and 99.18 ± 2.79 μm, respectively]. According to Three Way ANOVA, the main effect of preparation and material had no significant effect on the marginal adaptation where [F = 0.426, *p* = 0.516 and F = 1.242, *p* = 0.269, respectively]. However, the cementation was significantly increasing the marginal gap after cementing the crowns [F = 113.671, p = < 0.0001]. The interaction between the three factors did not show any significant effect on marginal adaptation (Table 4).

**Table 3:** Descriptive values of marginal adaptation (µm) among the study groups

		Subgroups			
		Va (*n* = 10)	Vb (*n* = 10)	MVa (*n* = 10)	MVb (*n* = 10)
Before cementation	Mean ± SD	93.18 ± 1.55	92.81 ± 1.66	92.99 ± 1.13	92.94 ± 2.02
	95% CI	92.06, 94.29	91.63, 94.00	92.18, 93.80	91.49, 94.38
After cementation and aging	Mean ± SD	99.72 ± 3.34	98.84 ± 2.65	99.18 ± 2.79	97.98 ± 3.66
	95% CI	97.33, 102.11	96.94, 100.74	97.18, 101.17	95.37, 100.60

n: Number of specimen

S.D.: Standard Deviation

CI: Confidence interval

**Table 4:** Three way ANOVA assessing the effect of preparation design and material on marginal adaptation

Variables	Mean Square	F test	*p* value
Preparation design	2.567	0.426	0.516
Material	7.738	1.242	0.269
Cementation and aging	708.288	113.671	< 0.0001*
Preparation x Material and aging	0.000	0.000	0.996
Preparation x Cementation and aging	2.224	0.357	0.552
Material x Cementation and aging	3.445	0.553	0.460
Preparation x Material x Cementation and aging	0.490	0.079	0.780
Corrected Model	103.549	16.618	< 0.0001*

*Statistically significant difference at p value < 0.05, Adjusted R squared = 0.581

*F* F for One way ANOVA test

p: *p* value for comparing between the studied groups

### Fracture resistance

Results of fracture resistance evaluation measured in Newton (N) are shown in (Table 5) and revealed that the preparation design has a considerable influence on the fracture resistance of monolithic crowns. The mean ± SD of fracture resistance was the highest among (MVb) subgroup (2123.58 ± 8.93 N) followed by (Vb) subgroup (1875.63 ± 64.84 N), followed by (MVa) subgroup (1064.94 ± 21.85 N) and the lowest mean ± SD value was obtained by (Va) subgroup (971.59 ± 47.47 N). Based on the two-way ANOVA test we found that the preparation design, the material, and their interaction effect had a significant effect on the fracture resistance where [F = 166.063, p value = < 0.0001, F = 5491.447, p value = < 0.0001 and F = 34.067, p value = < 0.0001, respectively] (Table 6). The pair-wise comparison revealed that the mean difference of the preparation designs (Modified vertical vs. Vertical) on the fracture resistance was (+ 170.65 N), while the mean difference of the material (zolid ht + vs. e.max) on the fracture resistance was (+ 981.34 N) (Table 7).

**Table 5:** Descriptive values of fracture resistance (N) among the study groups

	Subgroups			
	Va (*n* = 10)	Vb (*n* = 10)	MVa (*n* = 10)	MVb (*n* = 10)
Mean ± SD	971.59 ± 47.47	1875.63 ± 64.84	1064.94 ± 21.85	2123.58 ± 8.93
95% CI	937.63, 1005.54	1829.25, 1922.01	1049.31, 1080.58	2117.18, 2129.97

n: Number of specimen

S.D.: Standard Deviation

CI: Confidence interval

**Table 6:** Two way ANOVA assessing the effect of preparation design and material on fracture resistance

Variables	Mean Square	F test	*p* value
Preparation design	291221.051	166.063	< 0.0001*
Material	9630242.702	5491.447	< 0.0001*
Preparation x Material	59742.078	34.067	< 0.0001*
Corrected model	3327068.611	1897.192	< 0.0001*

Statistically significant difference at p value < 0.05, Adjusted R squared = 0.993

*F* F for One way ANOVA test

p: *p* value for comparing between the studied groups

**Table 7:** Pairwise comparison between preparation design and material

Variables		Mean difference
Preparation design	Modified vs. Vertical	+ 170.65
Material	Zirconia vs. e-max	+ 981.34

*Statistically significant difference at p value < 0.05

### Modes of fracture

**Fig. 7:**
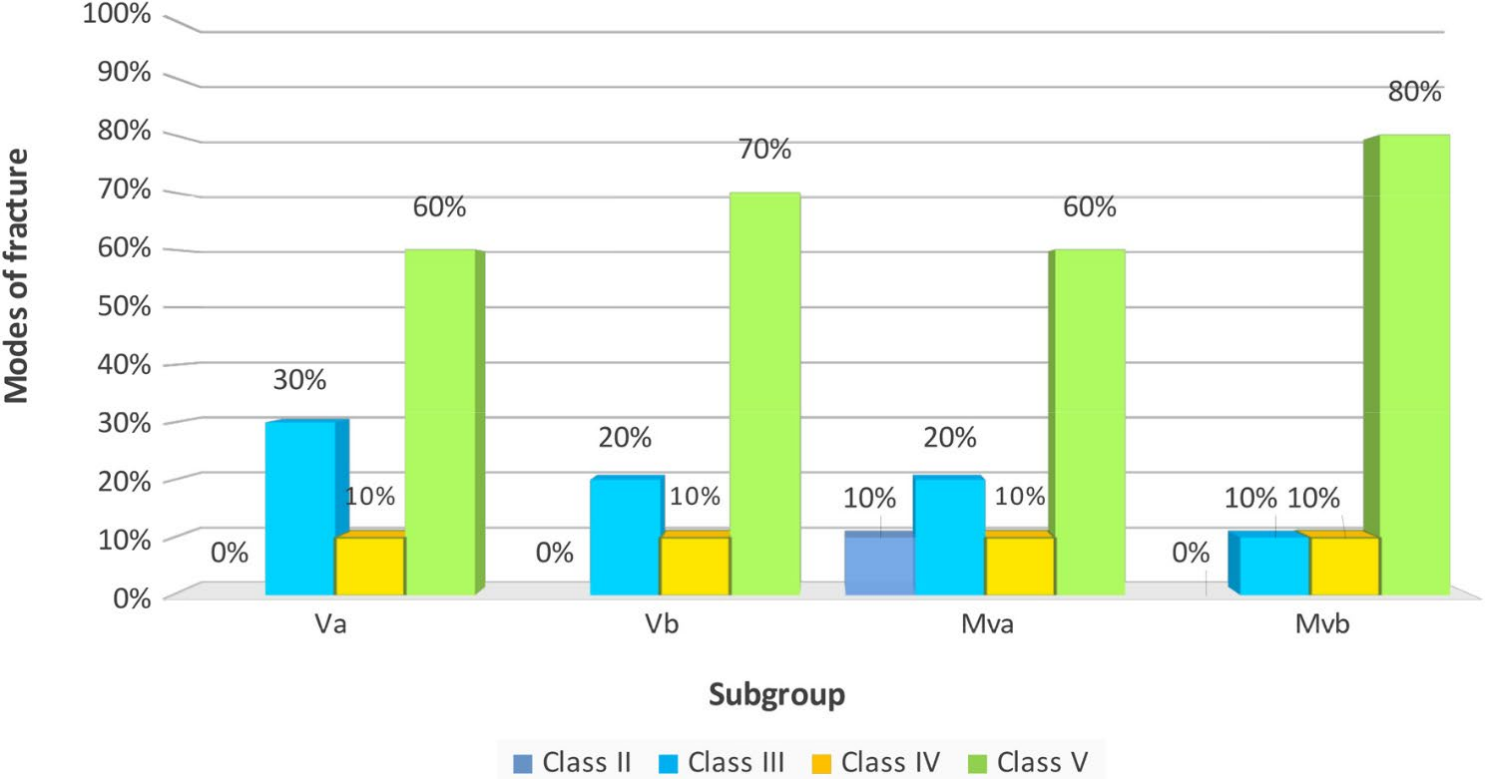
Chart showing comparison of mode of fracture of the four studied subgroups

**Fig. 8:**
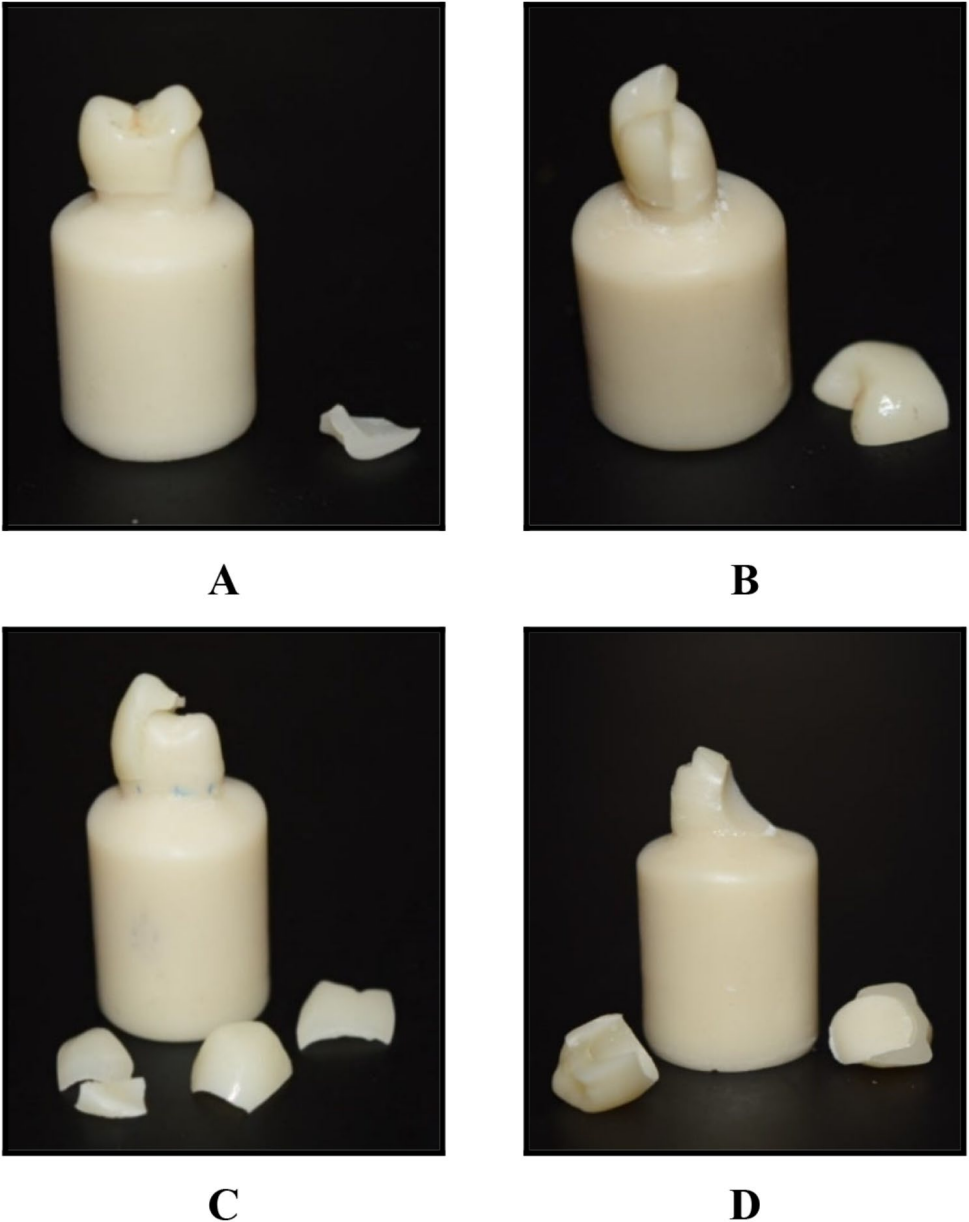
Modes of fracture (**A**) Mode II: less than half of the crown lost. (**B**) Mode III: half of the crown lost. (**C**) Mode IV: More than half of the crown lost. (**D**) Mode V: Severe fracture of the crown and tooth

The mode of fracture has no statistically significant difference among the four studied subgroups (*p* = 0.982). (Table 8; Figs. 7 and 8). In the (Va) subgroup (*n* = 10), 3/10 (30.00%) had mode III of fracture, 1/10 (10.00%) of mode IV of fracture, and 6/10 (60.00%) of mode V of fracture. In the (Vb) subgroup (*n* = 10), 2/10 (20.00%) had mode III of fracture, 1/10 (10.00%) of mode IV of fracture, and 7/10 (70.00%) of mode V of fracture. In the (MVa) subgroup (*n* = 10), 1/10 (10.00%) had mode II of fracture, 2/10 (20.00%) had mode III of fracture, 1/10 (10.00%) of mode IV of fracture, and 6/10 (60.00%) of mode V of fracture. In the (MVb) subgroup (*n* = 10), 1/10 (10.00%) had mode III of fracture, 1/10 (10.00%) of mode IV of fracture, and 8/10 (80.00%) of mode V of fracture.

**Table 8:** Comparison of modes of fracture of the four studied subgroups

	Subgroups			
Modes of fracture	Va (*n* = 10)	Vb (*n* = 10)	MVa (*n* = 10)	MVb (*n* = 10)
II	0 (0.00%)	0 (0.00%)	1 (10.00%)	0 (0.00%)
III	3 (30.00%)	2 (20.00%)	2 (20.00%)	1 (10.00%)
IV	1 (10.00%)	1 (10.00%)	1 (10.00%)	1 (10.00%)
V	6 (60.00%)	7 (70.00%)	6 (60.00%)	8 (80.00%)
Test of significance *p*	c^2^_(MC)(df=9)_ = 4.407 *p* = 0.982 NS			

n: Number of specimen

χ^2^ = Pearson Chi-Square

df = degree of freedom

MC = Monte Carlo

NS: Statistically not significant (*p* ≥ 0.05)

## Discussion

This in-vitro study evaluated the marginal adaptation and fracture resistance of lithium disilicate (e.max CAD) and zircon (zolid ht+) crown restorations fabricated by CAD/CAM technology with two preparation designs; vertical and modified vertical. The null hypothesis was partially accepted as there was no significant difference between the tested subgroups regarding the marginal adaptation neither before cementation nor after cementation and aging. On the other hand, the preparation designs used had a significant effect on fracture resistance of the studied subgroups.

For standardization, the two types of crown preparation were performed using a modified dental surveyor. For all specimens, a dual-cured universal adhesive resin cement was used to lute all the crown restorations to their corresponding epoxy resin dies that were used because of its dimension stability and its modulus of elasticity (11.8GPa) which is close to that of dentin (18GPa) [26]. CAD/CAM technology was chosen to manage thickness and anatomy, ensuring higher precision, consistency, and efficiency. Thermal cycling and cyclic loading were done to mimic the intraoral conditions by which all the studied crowns were survived. In this investigation, the number of thermocycling cycles was set to 5000 cycles at temperatures ranging from 5° ± 0.5℃ to 55° ± 0.5℃ with a 30 s dwell duration following the international organization of standardization recommendations for optimal aging [27].

One of the most crucial factors to consider is the cervical margin design as it can impact the marginal fit of the crown leading to recurrent caries and periodontal complications [28]. Furthermore, Wahsh et al. [28] declared that the marginal fit is the most relevant in crown evaluation and should be considered the most important. Assessment of the marginal fit of all crowns in four preselected locations before cementation and after cementation and aging were done by direct view method using a stereo microscope at a fixed magnification of 40x, this technique has the benefit of providing noninvasive, accurate, and repeatable measurements, making it useful for detecting the precision of fit of the entire specimen margin [29].

The mean marginal adaptation of all specimens that were tested in this present study was between 92.18 μm and 93.18 μm before cementation and between 97.98 μm and 99.72 μm after cementation and aging. These findings of all crowns manufactured with both preparations were within the clinically acceptable range (< 120 μm) [30]. In a study by Comlekoglu et al. [31], the feather-edge preparation had the highest marginal adaption, according to the findings. This was due to the fact that the more the restoration margin finishes with a sharp angle, the smaller the marginal gap, as previously explained by Shillingburg et al. [6].

Regarding the two different CAD/CAM ceramic materials used in the present study, many factors can impact their marginal fit such as scanning technique, software design, firing device and milling tools beside ceramic material liability to shrink after milling and sintering, for IPS e.max CAD restorations there is 0.2% linear shrinkage [32] and during the sintering process, zirconia exhibits around 20–25% linear shrinkage [33].

Following cementation and aging, it was revealed that the marginal fit had significantly changed. The increase in the resin-luting cement’s hydraulic pressure may be the cause of this. Additionally, the findings of the present study was in agreement with many studies that stated that cementation process increased the marginal gap of crown restorations [34–36].

Fracture resistance is one of the essential parameters to assess the long-term efficacy of CAD/CAM produced restoration [37]. The structural damage that accumulates throughout mastication may lead to the restoration’s fracture [38]. The results of this study showed that establishing the reverse shoulder modification had a significant effect on the improvement of the fracture resistance. This result could be related to the presence of more material thickness on the axial wall leading to more stress distribution.

The current study’s findings revealed that (MVb) subgroup showed the highest fracture resistance values followed by (Vb) subgroup followed by (MVa) subgroup, and the lowest results were with (Va) subgroup.

When discussing the fracture resistance of crowns with vertical and modified vertical preparation designs, factors such as material choice become crucial. The superior fracture resistance of the zirconia crowns with modified vertical preparation might be attributed to high mechanical properties and crack propagation resistance advantage of the zirconia restoration that might benefit from this preparation design as it allows better load and stress distribution. On the other hand, lithium disilicate crowns with vertical or modified preparation design showed less but still acceptable fracture strength values which were more than 900 N which is more than the maximum chewing force (700 N) of the healthy young people [28]. This suggested that the feather-edge preparation either with or without modification offers durable crowns that are satisfactory. Additionally, the feather edge preparation has biological advantages, such as preserving gingival and periodontal health by minimizing the amount of healthy tooth structure lost and preventing the margin of a crown restoration from being over contoured.

The findings of the present study are in accordance with Abdulazeez et al. [39] who reported that the zirconia crown restoration with a modified vertical preparation showed higher fracture resistance over those with vertical preparation. Another study by Jasim et al. [40] reported that zirconia crowns with feather edge preparation design with 1 mm occlusal thickness demonstrated greater fracture resistance than those with chamfer margin design with 0.5 mm occlusal thickness. Also Beuer et al. [41] examined the manner of load distribution for the feather-edge preparation; as the load increased, the zirconia coping carried the force to the axial walls without being restricted by the margin, concentrating the stress on the occlusal surface. Thus, occlusal thickness is the most essential factor since a decrease in occlusal thickness leads to poorer restorations. The occlusal thickness had a greater effect on the fracture resistance value than the cervical thickness [19, 42].

The majority of samples in the present study revealed an irreparable fracture of tooth or crown (mode V). This is due to the fact that, as compared to unprepared teeth, an increase in reduction in tooth preparation results in a decrease in fracture strength [3].

Several shortcomings were highlighted in the study, including: The study was performed in vitro with epoxy resin dies used as abutments instead of natural teeth that can influence the bonding and fracture resistance results. These characteristics differ from those of the intraoral environment. Additionally, the limited tested materials. Additional studies with more materials variety are recommended. Clinical studies and ongoing research are essential to validate the long-term success of crowns with modified vertical preparations.

## Conclusions

Within the limitations of this in vitro study, the following conclusions could be drawn:

- The marginal adaptation measurements either before cementation or after aging and cementation of lithium disilicate and zirconia crown restorations that were observed in this investigation were not affected with vertical and modified vertical preparation designs and were within clinically acceptable ranges.
- The fracture strength of monolithic zirconia and lithium disilicate crowns was significantly increased by the use of a modified vertical preparation with a reverse shoulder.
- The average fracture strength values of the monolithic zirconia and lithium disilicate crowns across all groups were greater than the premolar area maximal biting forces.
- Modified vertical preparation design could be a more conservative alternative to other margin designs with guarantees of high fracture resistance and acceptable marginal adaptation.

## Data Availability

The datasets generated and analyzed during the current study are available from the corresponding author on reasonable request.

## Abbreviations

mm: Millimeter
°: Degree
STL: Stereo-lithography
ED5x: 5-axis simultaneous dental mill
CAD/CAM: Computer-aided design & computer-aided manufacturing
>%: Percent
>°C: Centigrade degrees
min: Minute
sec: Second
>µm: Micrometer
GPa: Gigapascal
N: Newton
P: Probability
IBM SPSS: Software for advanced statistical analysis
